# Association between averaged intraoperative nociceptive response index and postoperative complications after lung resection surgery

**DOI:** 10.1093/icvts/ivac258

**Published:** 2022-10-10

**Authors:** Takuma Okamoto, Yuka Matsuki, Hiroki Ogata, Hiroai Okutani, Ryusuke Ueki, Nobutaka Kariya, Tsuneo Tatara, Munetaka Hirose

**Affiliations:** Department of Anaesthesiology and Pain Medicine, Hyogo Medical University School of Medicine, Nishinomiya, Japan; Department of Anaesthesiology and Reanimatology, Faculty of Medicine Sciences, University of Fukui, Fukui, Japan; Department of Anaesthesiology and Pain Medicine, Hyogo Medical University School of Medicine, Nishinomiya, Japan; Department of Anaesthesiology and Pain Medicine, Hyogo Medical University School of Medicine, Nishinomiya, Japan; Department of Anaesthesiology and Pain Medicine, Hyogo Medical University School of Medicine, Nishinomiya, Japan; Department of Anaesthesiology and Pain Medicine, Hyogo Medical University School of Medicine, Nishinomiya, Japan; Department of Anaesthesiology and Pain Medicine, Hyogo Medical University School of Medicine, Nishinomiya, Japan; Department of Anaesthesiology and Pain Medicine, Hyogo Medical University School of Medicine, Nishinomiya, Japan

**Keywords:** Morbidity, Nociception, Surgical invasiveness, Video-assisted thoracic surgery

## Abstract

**OBJECTIVES:**

Since postoperative complications, defined as Clavien–Dindo grade ≥II, correlate with long-term survival after lung resection surgery in patients with primary lung cancer, identification of intraoperative risk factors for postoperative complications is crucial for better perioperative management. In the present study, we investigated the possible association between intraoperative variables for use in anaesthetic management and Clavien–Dindo grade ≥II.

**METHODS:**

In this multi-institutional observational study, consecutive adult patients undergoing video-assisted thoracic surgery for primary lung cancer under general anaesthesia from March 2019 to April 2021 were enrolled. All patients were divided into 2 groups with Clavien–Dindo grade <II and ≥II. Uni- and multivariable analyses were performed to identify intraoperative risk factors.

**RESULTS:**

After univariable analysis between patients with Clavien–Dindo grade <II (*n* = 415) and ≥II (*n* = 121), multivariable analysis revealed higher averaged nociceptive response (NR) index during surgery (mean NR), male sex, lower body mass index, longer duration of surgery, higher blood loss and lower urine volume, as independent risk factors for postoperative complications. In sensitivity analysis, based on the cut-off value of mean NR for postoperative complications, all patients were divided into high and low mean NR groups. The incidence of postoperative complications was significantly higher in patients with high mean NR (*n* = 332) than in patients with low mean NR (*n* = 204; *P *<* *0.001).

**CONCLUSIONS:**

Higher mean NR, as intraoperative variables for use in anaesthetic management, is associated with the higher incidence of postoperative complications after primary lung cancer surgery.

**Figure:**
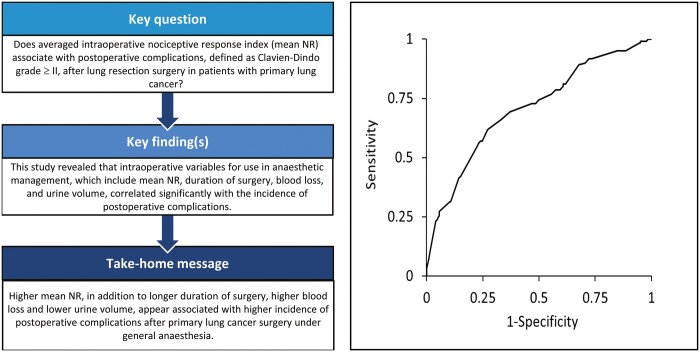

## INTRODUCTION

A higher incidence of postoperative complications after lung cancer surgery correlates with worse prognosis, including cancer recurrence and mortality [1–3]. Associations between postoperative complications (defined as Clavien–Dindo grade ≥II) and long-term survival after lung resection surgery have recently been reported in patients with primary lung cancer [4–6]. Although identification of intraoperative risk factors for postoperative complications is crucial for better perioperative management, intraoperative risk factors for postoperative complications with Clavien–Dindo grade ≥II have not been evaluated in detail for patients with primary lung cancer undergoing lung resection surgery.

The nociceptive response (NR) index, which is a dimensionless number between 0 and 1, was developed to quantitatively evaluate physiological responses caused by the balance between nociception induced by surgical stimuli and anti-nociception due to anaesthetic management in patients under general anaesthesia [7, 8]. NR values are calculated using 3 haemodynamic variables of heart rate (HR), systolic blood pressure (SBP) and perfusion index (PI). A higher NR index corresponds to the balance of physiological responses between nociception and anti-nociception inclining towards higher levels of nociception rather than anti-nociception, which induce higher surgical stress responses causing postoperative complications [9]. Although a higher mean NR index during surgery is reportedly associated with postoperative complications after gastrointestinal surgery [10, 11], associations between mean NR and postoperative complications have not been evaluated in patients with primary lung cancer undergoing lung resection surgery. To identify intraoperative risk factors for postoperative complications, we hypothesized that intraoperative variables for use in anaesthetic management, including mean NR index during surgery, would correlate with Clavien–Dindo grade ≥II in patients with primary lung cancer undergoing video-assisted thoracic surgery (VATS) under general anaesthesia in the present study.

## PATIENTS AND METHODS

### Ethics approval

This retrospective observational study was approved by the Ethics Committee of Hyogo Medical University (Ethics Committee number 3138; Chairperson Koichi Noguchi) on 4 March 2019. The requirement for written informed consent for study participation was waived by the institutional ethics committee. This study was conducted in accordance with the principles of the Declaration of Helsinki.

### Patients

Patients with primary lung cancer undergoing VATS for lung resection with curative intent from March 2019 to April 2021 at the surgical centres of Hyogo Medical University Hospital and University of Fukui Hospital were enrolled. All smokers quit smoking at least 1 month before surgery. Patients with age <19 years, and for whom serum concentrations of C-reactive protein (CRP) were not measured before and/or after surgery were excluded. Patients with conversion to thoracotomy during VATS procedure were also excluded.

### Data collection

To assess both the primary outcome of identifying intraoperative risk factors for Clavien–Dindo grade ≥II complications, and the secondary outcome of clarifying the association between mean NR index during surgery and Clavien–Dindo grade ≥II complications, we obtained perioperative data of age, sex, body mass index (BMI), American Society of Anesthesiologists Physical Status (ASA-PS), anaesthetic management, extent of lung resection (wedge resection, segmentectomy, lobectomy, bilobectomy and pneumonectomy), serum concentrations of CRP before and after surgery on postoperative day (POD) 1 and complications occurring within 30 days after surgery, classified using the Clavien–Dindo classification, from our institutional medical records. The Clavien–Dindo classification has been used to categorize postoperative complications into 5 grades: grade I, any deviation from the normal postoperative course without the need for pharmacological treatment or surgical, endoscopic and radiological interventions; grade II, complications requiring drug treatments other than those allowed for grade I complications; grade III, complications requiring surgical, endoscopic or radiological interventions; grade IV, life-threatening complications; and grade V, death [12]. In the present study, postoperative complications were defined as Clavien–Dindo grade ≥II.

### Surgical procedure and mean nociceptive response index during surgery

VATS was performed through the single-port or two-port approach with a small incision around 4 cm in the lateral decubitus position. NR index, which represents an objective value of the balance between nociception and anti-nociception under general anaesthesia, was calculated every 1 min during surgery from an equation that includes HR, SBP and PI, as follows [7, 8]:

$$\mathrm{NR}=\;-1+\frac{2}{1\;+e^{-\left(0.01HR+0.02S\mathrm{BP}-0.17PI\right)\;}}.$$

PI values were derived from the plethysmographic pulse wave amplitude via pulse oximetry. SBP values were obtained from a direct arterial pressure monitor. In Hyogo Medical University, the equation for NR index was installed on the anaesthesia information managing system (ORSYS; Philips Japan, Tokyo, Japan), and mean NR index from the start to the end of surgery in each patient was obtained using Vi-Pros data-search software (Dowell Co., Sapporo, Japan). Both NR index and mean NR index for each patient who underwent surgery at the University of Fukui Hospital were manually calculated using the equation for NR index.

### Perioperative anaesthetic management

No patients received premedication. Dexamethasone (3.3 mg) was injected intravenously before anaesthesia induction to prevent postoperative nausea and vomiting. General anaesthesia was induced with propofol, along with fentanyl and rocuronium, and then tracheal intubation with double-lumen tube was performed for one-lung ventilation. After anaesthesia induction, an arterial catheter was inserted into the radial artery contralateral to the side of surgery. Anaesthesia was maintained with propofol, in addition to fentanyl, rocuronium and continuous infusion of remifentanil. Doses of remifentanil and fentanyl were adjusted to maintain mean blood pressure within the range of ±20% of the pre-anaesthesia level. The dose of propofol was adjusted to maintain the bispectral index between 40 and 60. NR index was not used for anaesthetic management.

Multimodal analgesia was performed in all patients. Additional requirements for regional anaesthesia, including ultrasound-guided intercostal nerve block, ultrasound-guided thoracic paravertebral block and thoracic epidural block, were determined by the anaesthesiologists in charge based on experience and the preoperative comorbidities of patients. If thoracic epidural block was deemed appropriate by the anaesthesiologist in charge, a thoracic epidural catheter was inserted under infiltration anaesthesia using 1% mepivacaine with the patient in the lateral decubitus position before induction of general anaesthesia. Continuous infusion of 0.15% levobupivacaine at 4 ml h^−1^, along with fentanyl at 20 μg h^−1^ and bolus injection of 1% mepivacaine were administered epidurally during general anaesthesia. Postoperatively, patients continued to receive continuous epidural infusions of levobupivacaine and fentanyl at the same doses, with patient-controlled bolus injections of 3 ml, until POD3. Conversely, ultrasound-guided intercostal nerve block or ultrasound-guided thoracic paravertebral block, if deemed appropriate by the anaesthesiologist in charge, was performed with the patient in the lateral decubitus position after tracheal intubation. The nerve block needle was pulled out after confirming adequate spread of the local anaesthetic around the intercostal nerve or in the paravertebral space with bolus injection of 0.125–0.25% levobupivacaine. Success of the block was determined using ultrasonography. The total dose of levobupivacaine to be administered was confirmed to be below the maximum recommended dose before injection [13]. No catheter for continuous infusion was placed for these blocks.

After surgery, all patients who did not receive epidural analgesia received continuous administration of intravenous fentanyl at 25–50 μg h^−1^, along with oral administration of non-steroidal anti-inflammatory drugs, acetaminophen and tramadol, and transdermal fentanyl for postoperative analgesia.

### Sample size calculation

The sample size required for this observational study was calculated using PS Power and Sample Size Calculations version 3.0 software (Dupont WD and Plummer WD, Vanderbilt University Medical Center, Nashville, TN, USA). The calculation was performed assuming the probability of a type I error of 0.05 and a power of 0.8. Considering that the incidence of postoperative complications defined as Clavien–Dindo grade ≥II ranged from 26.8% to 44.4% after lung cancer surgery in previous studies [4, 14–16], we assumed that the probabilities of postoperative complications would be 25% and 40%, of which difference is clinically meaningful, in patients with low and high values of mean NR index, respectively. Based on these assumptions, the sample size was estimated as 165 in each group of patients with low or high mean NR index. In view of the potential for study dropouts, we enrolled 585 patients in this study.

### Statistics

Statistical testing was performed using JMS Pro version 14.2.0 (SAS Institute Inc., Cary, NC, USA) or IBM SPSS Statics 24 software (IBM Corp., Chicago, IL, USA). We divided all patients into 2 groups with postoperative complications of Clavien–Dindo grades <II and ≥II. Comparisons of variables between the 2 groups were performed using the chi-square test for categorical variables or Wilcoxon rank-sum test for numerical variables, which did not express normal distribution, and values of *P *<* *0.05 were considered to indicate statistical significance. Normality of data was assessed using the normal quantile plot.

Uni- and multivariable logistic regression analyses were performed to determine the correlation between intraoperative variables including mean NR index and postoperative complications. In univariable analysis, perioperative variables, which showed significant differences between the 2 groups (*P *<* *0.20), were selected as candidate variables for multivariable logistic analysis. Receiver-operating characteristic (ROC) curve analysis was used to identify the optimal cut-off for these variables. To exclude the confounding effects of mean NR index, we selected variables for which multicollinearity was not present between variables based on a variance inflation factor of ≥10. The results of logistic regression analysis are presented as odds ratios with 95% confidence intervals (CIs). To report the performance of multivariable logistic regression modelling, discrimination was assessed using ROC curve analysis [17].

### Sensitivity analysis

We also compared perioperative variables between groups of patients with mean NR less than the cut-off and greater than or equal to the cut-off for sensitivity analysis. Multivariable logistic regression analysis was performed to evaluate pre- and intraoperative variables for use in anaesthetic management, correlating with mean NR greater than or equal to the cut-off value. To report the performance of multivariable logistic regression modelling, discrimination was assessed using ROC curve analysis [17].

## RESULTS

**Figure 1:**
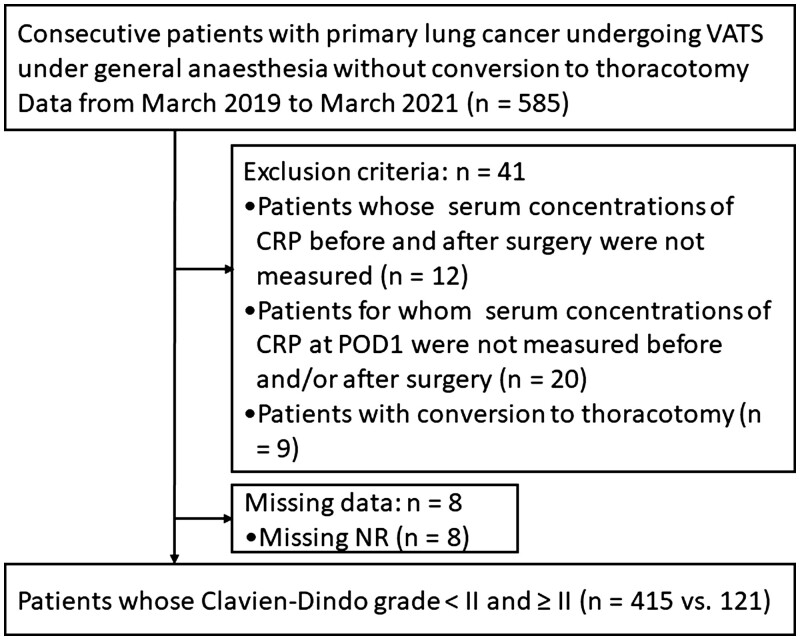
Consort diagram of patient recruitment. CRP: C-reactive protein; NR: nociceptive response; POD: postoperative day; VATS; video-assisted thoracic surgery.

Forty-one patients were excluded due to a lack of CRP data, and due to conversion to thoracotomy during VATS. Eight patients were also excluded due to missing NR data (Fig. 1). Table 1 shows perioperative variables in the remaining 536 patients enrolled in the present study (345 patients from Hyogo Medical University Hospital and 191 patients from the University of Fukui Hospital). In patients with Clavien–Dindo grade <II (*n* = 415), Clavien–Dindo grade I included postoperative pain with the need for opioids or pharmacological treatments for neuropathic pain (*n* = 178) and others. Postoperative complications, defined as Clavien–Dindo grade ≥II, occurred in 121 patents (22.6%). Clavien–Dindo grade II complications included surgical site infection (*n* = 15), severe postoperative pain (*n* = 12), pleural effusion (*n* = 10), liver dysfunction (*n* = 4), arrhythmia (*n* = 4), pneumonia (*n* = 3) and others (*n* = 29). Clavien–Dindo grade III and IV complications included air leak with the need for pleurodesis (*n* = 18), reoperation (*n* = 9), respiratory failure (*n* = 4), brain infarction (*n* = 2) and others (*n* = 11).

**Table 1:** Demographic characteristics and perioperative variables of patients

Perioperative variables, *n* = 536	
Preoperative variables	
Age (years), mean ± SD	69.7 ± 9.6
Female/male, *n* (%)	223/313 (41.6/58.4)
BMI (kg m^−2^), mean ± SD	22.9 ± 3.3
ASA-PS I/II/III/IV/V, *n* (%)	44/390/101/1/0 (8.2/72.8/18.8/0.2/0.0)
Elective surgery/emergency surgery, *n* (%)	533/3 (99.4/0.6)
Preoperative CRP level (mg dl^−1^), mean ± SD	0.28 ± 0.83
Intraoperative variables	
Extent of lung resection: wedge resection/segmentectomy, lobectomy or bilobectomy/pneumonectomy, *n* (%)	157/39/340/0 (29.3/7.3/63.4/0.0)
With/without regional anaesthesia, *n* (%)	199/337 (37.1/62.9)
NR, mean ± SD	0.835 ± 0.060
Continuous dose of remifentanil (μg kg^−1^ min^−1^), mean ± SD	0.145 ± 0.054
Total amount of fentanyl (μg kg^−1^ min^−1^), mean ± SD	0.024 ± 0.017
Total amount of rocuronium (mg kg^−1^ min^−1^), mean ± SD	0.008 ± 0.002
Duration of surgery (min), mean ± SD	142 ± 74
Duration of anaesthesia (min), mean ± SD	216 ± 77
Blood loss (ml kg^−1^ h^−1^), mean ± SD	0.24 ± 0.56
Urine volume (ml kg^−1^ h^−1^), mean ± SD	1.32 ± 1.09
Postoperative variables	
CRP level on POD1 (mg dl^−1^), mean ± SD	3.34 ± 2.62
Clavien–Dindo grade: none, I, II, III, IV, V, *n* (%)	56/359/77/41/3/0 (10.4/67.0/14.4/7.6/0.6/0.0)

ASA-PS: American Society of Anesthesiologists Physical Status; BMI: body mass index; CRP: C-reactive protein; NR: nociceptive response; POD: postoperative day; SD: standard deviation.

In univariable analyses, significant differences were seen in sex, BMI, mean NR index, continuous dose of remifentanil, total amount of fentanyl, duration of surgery, duration of anaesthesia, blood loss and postoperative CRP level on POD1 between patients with Clavien–Dindo grades <II and ≥II (Table 2).

**Table 2:** Univariable analyses between patients with Clavien–Dindo grades <II and ≥II

	Univariable analysis			ROC analysis	
	Clavien–Dindo grade <II *n* = 415 (77.4%)	Clavien–Dindo grade ≥II *n* = 121 (22.6%)	*P*-Value	AUC, *P*-value	Cut-off value
Age (years), mean ± SD	69 ± 9	70 ± 10	0.413	–	–
Female/male, *n* (%)	184/231 (44.3/55.7)	39/82 (32.2/67.8)	0.017*	–	–
BMI (kg m^−2^), mean ± SD	23.1 ± 3.4	22.1 ± 3.0	<0.001**	0.584, 0.004*	22.3
ASA-PS <III/≥III, *n* (%)	264/151 (63.6/36.4)	77/44 (63.6/36.4)	0.997	–	–
Elective surgery/emergency surgery, *n* (%)	413/2 (99.5/0.5)	120/1 (99.2/0.8)	0.655	–	–
Preoperative CRP level (mg dl^−1^), mean ± SD	0.22 ± 0.54	0.48 ± 1.41	0.002**	0.540, 0.008**	0.12
Extent of lung resection: wedge resection/segmentectomy, lobectomy or bilobectomy, *n* (%)	118/297 (28.4/71.6)	38/83 (31.4/68.6)	0.527	–	
With/without regional anaesthesia, *n* (%)	162/253 (39.0/61.0)	37/84 (30.6/69.4)	0.091	–	
NR, mean ± SD	0.831 ± 0.062	0.849 ± 0.049	0.004**	0.591, 0.004**	0.831
Continuous dose of remifentanil (μg kg^−1^ min^−1^), mean ± SD	0.141 ± 0.053	0.156 ± 0.054	0.009**	0.580, 0.010**	0.144
Total amount of fentanyl (μg kg^−1^ min^−1^), mean ± SD	0.023 ± 0.018	0.027 ± 0.014	0.033**	0.576, 0.045*	0.022
Total amount of rocuronium (μg kg^−1^ min^−1^), mean ± SD	0.008 ± 0.002	0.008 ± 0.002	0.592	–	–
Duration of surgery (min), mean ± SD	137 ± 63	158 ± 103	0.006**	0.544, 0.008**	221
Duration of anaesthesia (min), mean ± SD	210 ± 64	238 ± 106	<0.001**	0.571, <0.001**	232
Blood loss (ml kg^−1^ h^−1^), mean ± SD	0.18 ± 0.48	0.45 ± 0.75	<0.001**	0.681, 0.002**	0.08
Urine volume (ml kg^−1^ h^−1^), mean ± SD	1.36 ± 1.13	1.19 ± 0.89	0.176	0.526, 0.1768	2.17
CRP level on POD1 (mg dl^−1^), mean ± SD	3.09 ± 1.99	4.16 ± 4.00	<0.001**	0.565, <0.001**	6.26

Comparisons of 2 variables were performed using the Wilcoxon rank-sum test or chi-square test.

* Significant differences are defined at *P *<* *0.05.

** Significant differences are defined at *P *<* *0.01.

ASA-PS: American Society of Anesthesiologists Physical Status; AUC: area under the curve; BMI: body mass index; CRP: C-reactive protein; NR: nociceptive response; POD: postoperative day; ROC: receiver-operating characteristic; SD: standard deviation.

Postoperative complications were reportedly more frequent after segmentectomy than after wedge resection [18]. On the other hand, similar frequencies of postoperative complications were reported between segmentectomy and lobectomy [19]. Therefore, extents of lung resection were divided into 2 parts (wedge resection/segmentectomy, lobectomy or bilobectomy) in the present study and showed no significant difference between patients with Clavien–Dindo grades <II and ≥II (Table 2).

After identifying the optimal cut-off values for these variables using ROC curve analysis, we selected variables, for which multicollinearity was not present, to exclude the confounding effects of mean NR index. Selected variables for multivariable logistic regression analysis (*P* < 0.20) were male sex, BMI <22.3 kg m^−2^, mean NR index ≥0.831, continuous dose of remifentanil ≥0.144 μg kg^−1^ min^−1^, duration of surgery ≥221 min, blood loss ≥0.08 ml kg^−1^ h^−1^, urine volume <2.17 ml kg^−1^ h^−1^ and CRP level on POD1 ≥6.26 mg dl^−1^ (Tables 2 and 3).

**Table 3:** Multivariable logistic regression analysis of perioperative variables for Clavien–Dindo grade ≥II complications

Perioperative variables	Odds ratio [95% CI]	*P*-Value
Male sex	1.69 [1.06–2.72]	0.029*
BMI ≥22.3 kg m^−2^	0.53 [0.34–0.82]	0.005**
Preoperative CRP level ≥0.12 mg dl^−1^	–	0.557
Mean NR ≥0.831	2.31 [1.38–3.87]	0.001**
Continuous dose of remifentanil ≥0.144 μg kg^−1^ min^−1^	–	0.313
Duration of surgery ≥221 min	2.45 [1.30– 4.61]	0.005**
Blood loss ≥0.08 ml kg^−1^ h^−1^	1.74 [1.09–2.78]	0.021*
Urine volume ≥2.17 ml kg^−1^ h^−1^	0.37 [0.18–0.76]	0.007**
CRP level on POD1 ≥6.26 mg dl^−1^	–	0.649

* Significant differences are defined at *P *<* *0.05.

** Significant differences are defined at *P *<* *0.01.

BMI: body mass index; CI: confidence interval; CRP: C-reactive protein; NR: nociceptive response; POD: postoperative day.

**Figure 2:**
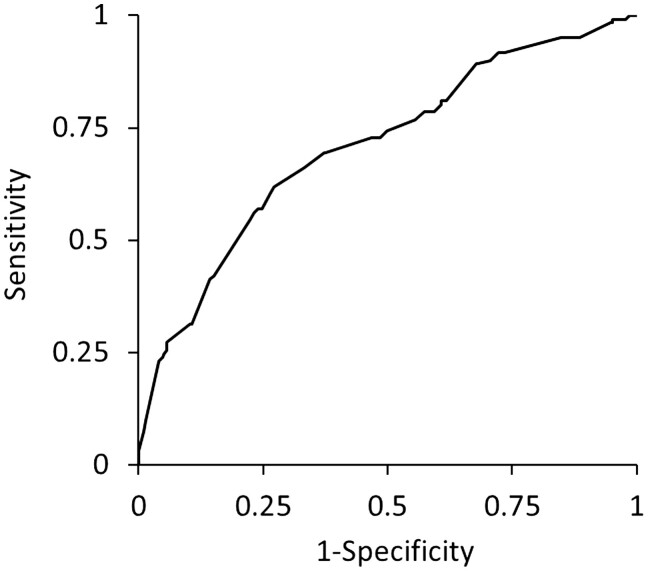
Receiver-operating characteristic curve for postoperative complications defined as Clavien–Dindo grade ≥II versus multivariate logistic regression model.

Thereafter, we performed multivariable logistic regression analysis using these variables, confirming that mean NR ≥0.831, in addition to male sex, BMI <22.3 kg m^−2^, duration of surgery ≥221 min, blood loss ≥0.08 ml kg^−1^ h^−1^ and urine volume <2.17 ml kg^−1^ h^−1^, were independent risk factors for postoperative complications (Table 3). ROC curve analysis showed that postoperative complications were significantly associated with these 6 variables (*P *<* *0.001), with an AUC of 0.708 [95% CI 0.651–0.759], and sensitivity and specificity of 0.620 and 0.728, respectively (Fig. 2).

### Sensitivity analysis

ROC curve analysis showed that mean NR was significantly associated with postoperative complications (*P *=* *0.004) at a cut-off mean NR index for postoperative complications of 0.831 (Table 2). According to this cut-off mean NR index for postoperative complications of Clavien–Dindo grades ≥II, patients were divided into 2 groups: those with mean NR <0.831 (*n* = 204) and ≥0.831 (*n* = 332) in the sensitivity analysis (Table 4). Although no significant differences in preoperative variables were seen between groups, significant differences were identified in the intraoperative variables of extent of lung resection (wedge resection/segmentectomy, lobectomy or bilobectomy), regional anaesthesia, higher doses of remifentanil and fentanyl and higher blood loss during surgery between patients with mean NR <0.831 and ≥0.831. In patients with mean NR ≥0.831, the incidence of postoperative complications was 28.3%, which was significantly higher than the 13.2% of incidence of complications seen in patients with mean NR <0.831 (*P *<* *0.001). CRP level on POD1 showed no significant difference between the 2 groups (*P *=* *0.728) (Table 4).

**Table 4:** Perioperative variables in patients with mean NR <0.831 and ≥0.831

	Mean NR <0.831 (*n* = 204)	Mean NR ≥0.831 (*n* = 332)	*P*-Value
Preoperative variables			
Age (years), mean ± SD	69 ± 10	70 ± 9	0.377
Female/male, *n* (%)	82/122 (40.2/59.8)	141/191 (42.5/57.5)	0.604
BMI (kg m^−2^), mean ± SD	23.2 ± 3.4	22.7 ± 3.2	0.057
ASA-PS <III/≥III, *n* (%)	140/64 (68.6/31.4)	201/131 (60.5/39.5)	0.059
Elective surgery/emergency surgery, *n* (%)	203/1 (99.5/0.5)	330/2 (99.4/0.6)	0.866
Preoperative CRP level (mg dl^−1^), mean ± SD	0.29 ± 0.84	0.26 ± 0.82	0.692
Intraoperative variables			
Extent of lung resection: wedge resection/segmentectomy, lobectomy or bilobectomy, *n* (%)	75/129 (36.8/63.2)	81/251 (24.4/75.6)	0.002**
With/without regional anaesthesia, *n* (%)	111/93 (54.4/45.6)	88/244 (26.5/73.5)	<0.001**
Continuous dose of remifentanil (μg kg^−1^ min^−1^), mean ± SD	0.121 ± 0.044	0.159 ± 0.055	<0.001**
Total amount of fentanyl (μg kg^−1^ min^−1^), mean ± SD	0.017 ± 0.014	0.028 ± 0.017	<0.001**
Total amount of rocuronium (μg kg^−1^ min^−1^), mean ± SD	0.008 ± 0.002	0.008 ± 0.002	0.355
Duration of surgery (min), mean ± SD	139 ± 74	144 ± 75	0.439
Duration of anaesthesia (min), mean ± SD	212 ± 75	219 ± 77	0.338
Blood loss (ml kg^−1^ h^−1^), mean ± SD	0.15 ± 0.36	0.30 ± 0.64	0.006**
Urine volume (ml kg^−1^ h^−1^), mean ± SD	1.28 ± 0.94	1.35 ± 1.17	0.494
Postoperative variables			
CRP level on POD1 (mg dl^−1^), mean ± SD	3.39 ± 2.44	3.30 ± 2.72	0.728
Clavien–Dindo grade <II/≥II, *n* (%)	177/27 (86.8/13.2)	238/94 (71.7/28.3)	<0.001**

Comparisons of 2 variables were performed using the Wilcoxon rank-sum test or chi-square test.

* Significant differences are defined at *P *<* *0.05.

** Significant differences are defined at *P *<* *0.01.

ASA-PS: American Society of Anesthesiologists Physical Status; BMI: body mass index; CRP: C-reactive protein; NR: nociceptive response; POD: postoperative day; SD: standard deviation.

Selected variables for multivariable logistic regression analysis (*P* < 0.20) were BMI, ASA-PS, extent of lung resection, regional anaesthesia, continuous dose of remifentanil, total amount of fentanyl and blood loss (Table 4). Multivariable logistic regression analysis using these intraoperative variables, for which the optimal cut-off values are shown in Table 2, revealed that higher extent of lung resection, no regional anaesthesia, continuous dose of remifentanil ≥0.144 μg kg^−1^ min^−1^, total amount of fentanyl ≥0.022 μg kg^−1^ min^−1^ and blood loss ≥0.08 ml kg^−1^ h^−1^ correlated significantly with mean NR ≥0.831 (Table 5). ROC curve analysis showed that mean NR ≥0.831 was significantly associated with these 3 variables (*P *<* *0.001), with an AUC of 0.749 [95% CI 0.704–0.789], the sensitivity of 0.678 and the specificity of 0.721.

**Table 5:** Multivariable logistic regression analysis of pre- and intraoperative variables for mean nociceptive response during surgery ≥0.831

Perioperative variables	Odds ratio [95% CI]	*P*-Value
BMI ≥22.3 kg m^−2^	–	0.442
ASA-PS ≥III	–	0.992
Extent of lung resection: wedge resection/segmentectomy, lobectomy or bilobectomy, *n* (%)	1.89 [1.23–2.92]	0.004**
With regional anaesthesia	0.58 [0.35–0.96]	0.035^*^
Continuous dose of remifentanil ≥0.144 μg kg^−1^ min^−1^	1.94 [1.26–2.99]	0.003**
Total amount of fentanyl ≥0.022 μg kg^−1^ min^−1^	2.48 [1.60–3.85]	<0.001**
Blood loss ≥0.08 ml kg^−1^ h^−1^	1.84 [1.21–2.81]	0.005**

^*^ Significant differences are defined at P < 0.05.

** Significant differences are defined at *P *<* *0.01.

ASA-PS: American Society of Anesthesiologists Physical Status; BMI: body mass index; CI: confidence interval.

## DISCUSSION

Intraoperative variables for use in anaesthetic management, including mean NR index during surgery ≥0.831, duration of surgery ≥221 min, blood loss ≥0.08 ml kg^−1^ h^−1^ and urine volume <2.17 ml kg^−1^ h^−1^, showed significant associations with postoperative complications of Clavien–Dindo grade ≥II in the present study. Although reported intraoperative risk factors for postoperative complications after lung cancer surgery include higher levels of surgical invasion as seen with thoracotomy compared to VATS or robot-assisted thoracic surgery, large tumour size, tumour location, longer duration of surgery and higher blood loss [16, 21, 22], to our knowledge, this study is the first to show intraoperative variables for use in anaesthetic management as risk factors for Clavien–Dindo grade ≥II in patients with primary lung cancer.

The incidence of postoperative complications was significantly higher in patients with mean NR index ≥0.831 than in patients with mean NR index <0.831 in the present study. Higher mean NR index represents a higher degree of surgical invasiveness [23], which is reportedly one of the risk factors for postoperative complications after lung cancer surgery [16, 20, 21]. Since a higher level of surgical invasion causes greater surgical stress responses in terms of sympathetic activity, stress hormone release and inflammation and eventually induces postoperative complications [24–26], the significant correlation of higher mean NR index with postoperative complications makes sense in the present study.

Extent of surgery, no regional anaesthesia, intraoperative variables of continuous dose of remifentanil ≥0.144 μg kg^−1^ min^−1^, total amount of fentanyl ≥0.022 μg kg^−1^ min^−1^ and blood loss ≥0.08 ml kg^−1^ h^−1^ correlated significantly with mean NR index ≥0.831 in the present study. The number of patients undergoing segmentecomy, lobectomy or bilobectomy was significantly higher in patients with mean NR index ≥0.831 than that in patients with mean NR index <0.831. In patients with mean NR index <0.831, both remifentanil and fentanyl usage during general anaesthesia were significantly lower than those in patients with mean NR index ≥0.831. Although regional anaesthesia was not identified as a risk factor for postoperative complications in the present study, intraoperative control of NR values using multimodal analgesia using low-dose opioids with regional anaesthesia might have potential benefits in suppressing postoperative complications after primary lung cancer surgery with curative intent. Nociception monitors (e.g., NR index, the nociception level index) reportedly represented the balance between nociception and anti-nociception during VATS [27, 28]. In addition, several other nociception monitors have been used during thoracic surgery [29]. Therefore, a randomized control study is required to examine the effects of nociception monitor-guided multimodal analgesia during general anaesthesia on postoperative complications in the future.

Preoperative variables of male sex and BMI <25 kg m^−2^ were selected as independent risk factors for postoperative complications in the present study. Previous studies have also reported male sex and low nutritional and immunological status, in addition to older age, higher ASA-PS grade, concomitant asthma or pulmonary disease, low percentage of forced expiratory volume in 1 s and current smoking, as preoperative risk factors for postoperative complications after lung cancer surgery [14–16, 20, 21, 30, 31]. In previous studies investigating the relationship between sex and postoperative complications after lung cancer surgery, male patients exhibited higher postoperative morbidity and mortality. Older age in male patients, higher incidences of chronic comorbidities and a history of smoking in male patients, and differences in tumour histology were reported as probable causes for the sex differences in postoperative outcomes [24, 32]. Another study showed that low nutritional and immunological status as assessed by the prognostic lymphocyte count calculated using serum albumin levels and peripheral lymphocyte counts predicts postoperative complications after lung cancer surgery [14]. Since low nutritional status correlates with low BMI, those results from previous studies support the present findings that both male sex and low BMI correlate significantly with postoperative complications.

### Limitations

One limitation of this study was the observational design. Another limitation was that the tumour, node, metastasis classification for lung cancer to characterize the extent of disease, which might correlate with surgical invasiveness, was not involved in the preoperative variables for analysis. In addition, we did not consider results of preoperative pulmonary function tests (e.g., forced expiratory volume in 1 s, diffusing capacity of lung for carbon monoxide) as risk factors for postoperative complications. Although there is a controversy on whether these pulmonary function tests would correlate with postoperative complications after lung resection [16, 20, 22], further investigation to evaluate the effects of NR-guided anaesthetic management on postoperative complications and long-term survival is required in a randomized-controlled manner in patients with the same tumour, node, metastasis classification while considering pulmonary function tests in the future.

## CONCLUSION

Intraoperative variables used in anaesthetic management, including higher mean NR index, longer duration of surgery, higher blood loss and lower urine volume, are likely associated with a higher incidence of postoperative complications after primary lung cancer surgery under general anaesthesia.

## Data Availability

The data underlying this article will be shared on reasonable request to the corresponding author.

## ABBREVIATIONS

ASA-PS: American Society of Anesthesiologists Physical Status
BMI: Body mass index
CIs: Confidence intervals
CRP: C-reactive protein
HR: Heart rate
NR: Nociceptive response
PI: Perfusion index
POD: Postoperative day
ROC: Receiver-operating characteristic
SBP: Systolic blood pressure
VATS: Video-assisted thoracic surgery
